# HLA-G Genotype/Expression/Disease Association Studies: Success, Hurdles, and Perspectives

**DOI:** 10.3389/fimmu.2020.01178

**Published:** 2020-07-08

**Authors:** Giada Amodio, Silvia Gregori

**Affiliations:** Mechanisms of Peripheral Tolerance Unit, San Raffaele Telethon Institute for Gene Therapy (SR-Tiget), IRCCS San Raffaele Scientific Institute, Milan, Italy

**Keywords:** HLA-G, immune regulation, autoimmune diseases, pregnancy, cancer, HLA-G polymorphisms

## Abstract

The non-classical HLA-G is a well-known immune-modulatory molecule. In physiological condition, HLA-G surface expression is restricted to the maternal–fetal interface and to immune-privileged adult tissues, whereas soluble forms of HLA-G are detectable in various body fluids. HLA-G can be *de novo* expressed in pathological conditions including tumors, chronic infections, or after allogeneic transplantation. HLA-G exerts positive effects modulating innate and adaptive immune responses and promoting tolerance, or detrimental effects inducing immune escape mechanisms. HLA-G locus, in contrast to classical HLA class I gene, is highly polymorphic in the non-coding 3′ untranslated region (UTR) and in the 5′ upstream regulatory region (5′ URR). Variability in these regions influences HLA-G expression by modifying mRNA stability or allowing posttranscriptional regulation in the case of 3′ UTR or by sensing the microenvironment and responding to specific stimuli in the case of HLA-G promoter regions (5′ URR). The influence of genetic variations on the expression of HLA-G makes it an attractive biomarker to monitor disease predisposition and progression, or response to therapy. Here, we summarize the current knowledge, efforts, and obstacles to generate a general consensus on the correlation between HLA-G genetic variability, protein expression, and disease predisposition. Moreover, we discuss perspectives for future investigation on HLA-G genotype/expression in association with disease predisposition and progression.

## Introduction

HLA-G, a non-classical HLA class I molecule, was first described to play a critical role in maintaining fetal–maternal tolerance (1). Later, it has been shown that HLA-G modulates innate and adaptive immune responses and promotes tolerance in different clinical settings. HLA-G function is favorable during pregnancy or after transplantation since it protects from rejection, and in autoimmune disease as it prevents autoreactive responses, or it is detrimental when expressed by tumors or during chronic infections, inducing immune escape mechanisms (2).

Because the HLA-G gene has a limited number of polymorphisms within the coding region, relatively few distinct molecules are coded. Nevertheless, seven different HLA-G isoforms have been described: four membrane-bound (HLA-G1 to -G4), and three soluble (HLA-G5 to -G7) (3–5).

The magnitude of HLA-G gene and protein expression is controlled by polymorphisms in the promoter [5′-upstream regulatory region (5′ URR)] and in the 3′ untranslated region (3′ UTR), and several association studies between these polymorphic sites and disease predisposition, response to therapy, and/or HLA-G protein expression have been reported. However, results from these studies often have been weak and inconclusive (6–10). Here, we summarize efforts to generate a general consensus on the correlation between HLA-G genetic variability, protein expression, and disease predisposition. Moreover, we highlight and discuss limits hampering the possibility to define a unique framework in the correlation between HLA-G genetic and disease predisposition or HLA-G genetic and protein expression, or HLA-G genetic/protein expression and disease predisposition.

## HLA-G Haplotypes

The HLA-G gene has 74 alleles encoding for 24 different full-length proteins, and four null alleles encoding for truncated form of the protein (IPD78/IMGT/HLA; March 2020). HLA-G locus, similar to other classical HLA class I locus, is composed of eight exons and seven introns, but it presents a stop codon in exon 6, resulting in a short cytoplasmic tail (4) and in an extended 3′ UTR, mainly composed by the exon 8 (11). Since HLA-G discovery in 1987 (12), HLA-G locus has been accurately analyzed, and the variability detected at the HLA-G regulatory regions (e.g., 3′ UTR and 5′ URR) is relatively higher than that observed in the coding region (13, 14).

The first identified and most studied polymorphism of the HLA-G locus is a 14-base-pair insertion/deletion (14-bp INS/DEL) landing in the 3′ UTR (15). More detailed and large genetic studies identified 16 additional single-nucleotide polymorphisms (SNPs) in the HLA-G 3′ UTR, of which only nine—the 14-bp INS/DEL polymorphism, +3003 C/G, +3010 G/C, +3027 C/A, +3035 C/T, +3142 C/G, +3187 A/G, +3196 C/G, and +3227 G/A—were recognized as true polymorphisms (13). The discovery that some of these polymorphisms are in strong linkage disequilibrium allowed the identification of 41 3′ UTR haplotypes, designated from UTR-1 to UTR-41, with only nine UTRs accounting for more than 95% of all haplotypes worldwide (13, 14, 16, 17).

The HLA-G locus presents also several variations in the 5′ URR and in the coding region. SNPs in these regions are in linkage disequilibrium, and a limited number of haplotypes, clustering in few families, have been identified and studied, alone or in combination with 3′ UTR alleles (18–20). In detail, the analysis of 35 SNPs within the 5′ URR revealed 64 different haplotypes (named PROMO), of which only nine representing the 95% of alleles worldwide, clustering in four major groups (PROMO 010101, 010102, 0103, and 010104) (13, 20, 21). Similarly, 81 variations were identified in the coding region, the majority landing in introns, arranging in 93 different haplotypes, of which only 11 having a frequency higher than 1% (13), including a null allele G^*^0105N encoding for a non-functional protein (22, 23). Interestingly, when the 5′ URR, the coding region, and the 3′ UTR haplotypes have been combined in the “extended haplotypes,” it was clear that, also in this case, among the 200 haplotypes identified, the majority of them was scarcely represented (13). Moreover, 5′ URR, coding region, and 3′ UTR haplotypes are in linkage disequilibrium; thus, a given PROMO haplotype is preferentially associated with one specific 3′ UTR and coding region haplotype (11, 20, 21).

## HLA-G Genetic Footprint and Correlation With Disease Course

HLA-G protein levels can be associated with specific genotypes; thus, a number of studies have been performed trying to correlate HLA-G haplotypes with disease susceptibility and morbidity or to use them as a predictive factor for response to therapy or in transplantation outcome (24).

The most studied HLA-G variation is the 14-bp INS/DEL polymorphism. In the field of pregnancy, there is a quite good consensus on the association of the 14-bp INS/INS genotype with recurrent pregnancy loss (RPL); however, a number of caveats have been identified in these correlations, including the heterogeneity of the studies, the sample characteristics, and non-comparable measure of the genotypes (23–25). A recent meta-analysis considering only women of European countries not only corroborated the association of 14-bp INS/INS genotype and RPL, but also highlighted and confirmed that the discrepancies observed in previous studies could be due to ethnic diversity of the cohort analyzed (26).

In autoimmunity and cancer, results on the association of 14-bp INS/DEL genotypes with disease development or with response to therapy reported contradictory and/or inconclusive results. In the context of type 1 diabetes (T1D), the analysis revealed the association of the 14-bp DEL/DEL genotype with the development and the early onset of the disease (27, 28). Conversely, in Crohn's disease, a high frequency of the 14-bp INS allele has been associated with an increased risk of early disease onset (29). A meta-analysis performed considering 11 case–control studies from different autoimmune diseases showed the absence of a direct correlation between 14-bp INS/DEL genotypes and susceptibility to autoimmune diseases, including systemic lupus erythematosus, rheumatoid arthritis multiple sclerosis, ulcerative colitis, Crohn's disease, idiopathic dilated cardiomyopathy, pemphigus vulgaris, and non-segmental vitiligo (7). The discrepancies observed might be attributed to the etiological mechanisms of the autoimmune disease in which gene-to-gene and gene-to-environment interactions are involved (see below), to the age of disease onset, and to the sample sizes.

A meta-analysis carried out to solve the 14-bp INS/DEL genotype association with cancer, including a large sample size, a wide variety of cancer types, and a more diverse sample population, overall revealed that HLA-G 14-bp INS/DEL polymorphism is significantly associated with the cancer susceptibility (30). However, these results are inconsistent with previous meta-analysis (8, 9, 24, 31), which concluded the absence of relationship between the HLA-G 14-bp INS/DEL polymorphism and the risk of cancer. Despite the positive correlation between 14-bp INS/DEL genotype and cancer susceptibility, some weaknesses of the latter study, linked to sample size, ethnicity, types of cancer, and sources of controls, have been reported. Indeed, stratified analysis accounting for the abovementioned variables failed to find a significant risk association (30).

In the context of allogeneic hematopoietic stem cell transplantation (HSCT), it has been shown that patients with better outcome carried 14-bp DEL/DEL and 14-bp INS/DEL genotypes, suggesting 14-bp INS/DEL genotype as potential biomarker of transplantation outcome (32, 33). Overall, the association between 14-bp INS/DEL genotypes and disease susceptibility provided some positive, but not conclusive, association. This might be attributed to a number of reasons, including high variability in the etiology of the diseases analyzed and to the relative limited number of patients enrolled in single correlation studies.

The discovery of 3′ UTR haplotypes and genotypes prompted investigators to reconcile the heterogeneity of the results obtained in studies on the association between 14-bp INS/DEL and diseases. We and other groups reported the protective role of specific UTRs in preventing RPL, with UTR-1, UTR-3, and UTR-4 present at low frequency in women with RPL (34–36). These studies indicate that analysis of 3′ UTR provided an improvement beyond the use of 14-bp INS/DEL genotypes in the association with pregnancy. UTR-1, UTR-3, and UTR-4 indeed contain 14-bp DEL, but they differ from other UTRs for additional specific SNPs (13, 14, 16, 17). Similar results were reported in T1D, showing that UTR-3 is present at low frequency in patients (37). In the context of allogeneic HSCT, only a weak association of the UTR-2, containing the 14-bp INS, with protection from acute graft-versus-host disease was reported, and the authors indicated that the study of the entire 3′ UTR, copamred to the sole analysis of the 14-bp INS/DEL, did not improve the prediction of transplant outcome (38).

Thus, far, few groups have investigated the impact of polymorphisms landing in the PROMO or the coding region of HLA-G with disease predisposition, and the most significant association has been found with the G^*^0105N null allele that is present with high frequency in RPL (19, 39, 40) and preeclampsia (41, 42). In celiac disease (CD), starting from the demonstration that 14-bp INS confers an increased risk factor for the disease in conjunction with the HLA-DQ2.5 (43), an extended analysis, including polymorphisms located in the PROMO and 3′ UTRs, showed the association of the haplotype containing PROMO 010102a and UTR-2 with CD susceptibility (44).

In conclusion, these results indicate that the correlation between HLA-G genotypes, and specifically those containing the 14-bp INS allele, and pregnancy loss or susceptibility to specific types of cancer has been achieved. Drawing conclusion on the association between HLA-G genotypes and susceptibility for other diseases is highly difficult because of the heterogeneity of the pathogenesis of the diseases analyzed, the age of disease onset, the cohorts of patients and controls used, and, in some cases, the limited number of case–control included in the study, disabling the statistical analysis.

## Association HLA-G Genotype/Phenotype

Several groups integrated the knowledge on HLA-G genetic with the detection of the HLA-G protein, both as soluble (s) (HLA-G5 and shed-HLA-G1) and as membrane-bound (HLA-G1) form, with the aim to use HLA-G as biomarker of disease predisposition and progression, or response to therapy.

In 2001, Rebmann et al. (45) reported the first evidence that HLA-G genotype influences the amount of sHLA-G in circulation. The study performed in healthy individuals defined some alleles (G^*^01013 and G^*^0105N) being associated with low levels, and other (G^*^01041) with high levels of sHLA-G. Later, the identification of HLA-G 3′ UTR polymorphic sites prompted investigators to correlate HLA-G genetic variation at 3′ UTR and protein expression. The presence of the 14-bp INS allele has been associated with lower HLA-G production for most HLA-G5 and shed-HLA-G1 in plasma or serum, and HLA-G1 in trophoblast samples [reviewed in Carosella et al. (2)]. However, the presence of the 14-bp sequence leads to alternative splicing and the generation of a more stable mRNA associated with high HLA-G1 expression in trophoblast cell lines (46).

Correlation studies performed in autoinflammatory and autoimmune diseases revealed that the 14-bp INS allele was associated with low plasma levels of sHLA-G in Crohn's disease patients (29). In relapsing–remitting multiple sclerosis, the 14-bp DEL/DEL genotype correlated to high levels of HLA-G in cerebrospinal fluid; however, it did not associate with disease duration and clinical symptoms (47). On the same line, in chronic lymphocytic leukemia, the 14-bp DEL allele has been associated with high levels of sHLA-G and HLA-G1, but only sHLA-G correlated with patient survival, possibly as a consequence of the high metalloproteinase activity involved in shedding HLA-G1 (48). A comprehensive analysis, performed to correlate HLA-G genotype/phenotype and disease outcome, showed a link between the 14-bp INS/INS genotype with pretransplant low sHLA-G levels and severe adverse events after HSCT (49). Similarly, the 14-bp DEL haplotype correlated with high sHLA-G serum levels and reduced episodes of heart transplant rejection (50). Despite the variety of the disease investigated, 14-bp INS and DEL alleles have been confirmed to be associated with low and high HLA-G, respectively, and in most cases high HLA-G with positive outcome of the disease.

More recently, investigators have studied the correlation of HLA-G 3′ UTR and protein expression. Haplotypes and diplotypes containing UTR-1 have been associated with high levels of plasma sHLA-G, those containing UTR-5 and UTR-7 with low levels of sHLA-G, and finally, alleles containing UTR-2, UTR-3, UTR-4, and UTR-6 with medium levels of sHLA-G (51). These results were confirmed in other biological fluids as the highest levels of HLA-G in seminal plasma have been detected in the presence of homozygosis for UTR-1 and UTR-3 (52). Our group defined the association of specific UTRs with the expression of HLA-G1 in a specific subset of tolerogenic DC, termed DC-10, inducible *in vitro* in the presence of IL-10 (53) and present *in vivo* (54). We showed higher frequency of UTR-2, UTR-5, and UTR-7 haplotypes and diplotypes in donors with DC-10 expressing low HLA-G1 and of UTR-3 in donors expressing high HLA-G1 (55). More recently, we confirmed that the UTR-3 haplotype is associated with high levels of HLA-G1 on circulating DC-10 (Amodio et al., submitted).

In conclusion, these results indicate a general consensus on the association between 14-bp INS and DEL allele and low and high expression of HLA-G, either soluble or membrane-bound isoforms, respectively. However, the 14-bp INS allele encodes for a transcript with a 92-bp deletion leading to a more stable mRNA fragment than that generated by the 14-bp DEL (56), suggesting that 14-bp INS might be also associated with high levels of HLA-G expression. Correlation studies including additional variations in the 3′ UTR improved the correlation between HLA-G genetic and protein expression partially solving the mRNA stability issue. Moreover, HLA-G protein expression is driven by genetic variations in the 3′ UTRs, but also by those landing in the promoter region; thus, variability of the microenvironment associated with specific disease could affect the HLA-G protein expression.

## Intracellular and Extracellular Mechanisms Regulating HLA-G Expression

**Figure 1:**
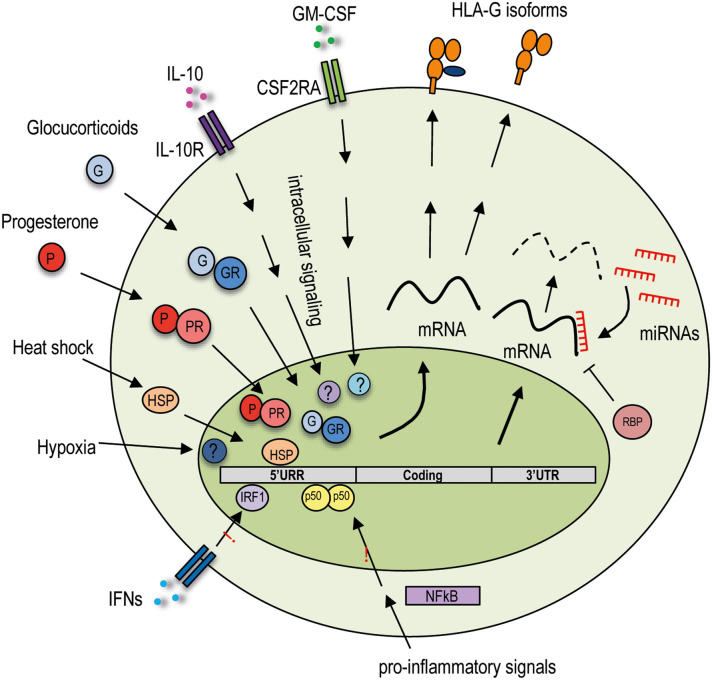
Extracellular and intracellular regulatory mechanisms of HLA-G expression. Variability in the HLA-G promoter region influences HLA-G expression by sensing and responding to the extracellular signals. Variations in the 3′ UTR region may modify mRNA stability or allow posttranscriptional regulation. HLA-G is not responsive to proinflammatory signals acting on the NF-κB pathway and to IFN-mediated stimulation. The HLA-G promoter region is unique among the HLA class I genes as it interacts with specific transcription factors activated by extracellular stimuli induced by hypoxia and heat shock, hormones such as glucocorticoids and progesterone, and cytokines including IL-10 and GM-CSF. HLA-G expression is posttranscriptionally regulated by genetic variations in the 3′ UTR, which contain several target sites for miRNAs and can bind specific RNA-binding proteins. These different regulations concur in the induction or inhibition of the expression of the HLA-G protein, which by alternative splicing of the mRNA can be produced in different isoforms: membrane-bound or soluble. 5′ URR, 5′ upstream regulatory region; 3′ UTR, 3′ untranslated region; CSF2RA, colony-stimulating factor 2 receptor subunit alpha; IL-10R, IL-10 receptor; IFNs, interferons; GR, glucocorticoid receptor; PR, progesterone receptor; HSP, heat shock protein, IRF-1, interferon regulatory factor 1; NF-κB, nuclear factor κ-light-chain-enhancer of activated B cells; RBP, RNA-binding proteins; miRNAs, microRNAs.

Genetic variations in the 3′ UTR, which contain several target sites for microRNAs (miRNAs), regulate at post-transcriptional level the HLA-G expression. Being miRNA cell-specific, this regulation may affect the expression of HLA-G at cell and tissue levels. Six miRNAs have been reported to regulate HLA-G expression: miR-148a, miR-148b, miR-152, miR-133a, miR-628-5p, and miR-548q (57). The direct effect of these miRNAs in HLA-G protein expression has been mainly demonstrated *in vitro*, using cell lines (Figure 1). However, several indirect evidences prompted investigators to correlate the presence of specific miRNA with HLA-G protein expression *in vivo*. In placenta, miR-148a and miR-152 are poorly expressed, whereas the HLA-G mRNA levels are high (58). Since miR-148a and miR-152 down-regulate HLA-G1 protein expression in cell lines, a possible inverse relationship between these molecules in placenta has been postulated (58). Similarly, miR-133 reduced HLA-G protein expression in trophoblast cell lines (59), and its low expression in primary colorectal cancer samples, in which the HLA-G levels are high (60), suggested a possible inverse correlation of these molecules. As anticipated avobe, thus far direct evidence of the role of specific miRNA on the expression of HLA-G *in vivo* is scanty.

An additional layer of posttranscriptional regulation of HLA-G protein expression is mediated by a specific RNA-binding protein (RBP) (Figure 1), the heterogeneous nuclear ribonucleoprotein R (HNRNPR), which binds the 3′ UTR of the transcripts, stabilizes them, and allows HLA-G1 expression in transduced cell lines (61). More recently, a distinct and unique region in the 3′ UTR of HLA-G has been identified, but neither miRNAs nor RBPs seem to bind to this site and to control HLA-G1 expression in cell lines (62). Thus, additional studies are warranted to define the relevance of this sequence into the regulation of HLA-G protein expression *in vivo*.

The HLA-G protein expression is regulated not only by allelic variability and posttranscriptional regulation, but also by specific regulatory regions present in the HLA-G promoter. Nucleotide variability in the promoter region may indeed influence HLA-G protein levels by modifying transcription factor–binding affinity. In the HLA-G locus, the enhancer A (EnhA) region, which allows the interaction with nuclear factor k-light-chain-ehnancer of activated B cells (NF-κB) family of transcription factors, binds only p50/p50 homodimers (63). Moreover, the binding of interferon regulatory factor 1 and 2 (IRF-1 and IRF-2) to interferon-stimulated response element in the HLA-G promoter is not present in HLA-G (64, 65). The presence of this unique HLA-G promoter region indicates that HLA-G expression is not influenced by NF-κB or by interferon α (IFN-α), IFN-β, and IFN-γ (Figure 1).

Despite the unresponsiveness to interferons, HLA-G transcriptional rate is increased by a number of anti-inflammatory cytokine and mediators, as the HLA-G promoter region presents specific regulatory elements. The presence in the HLA-G promoter of a heat shock element allows response to heat shock proteins (HSP) (Figure 1), essential for regulating the state of intracellular folding, assembly, and translocation of proteins, potent modulators of the immune responses, and necessary for placental development. HLA-G transcription is induced upon heat shock in tumor cell lines, *via* the heat shock transcription factor 1; however, the consequent HLA-G protein expression has not been investigated (66).

Glucocorticoids (dexamethasone) and progesterone, a hormone fundamental for endometrium maintenance and embryo implantation, increase the secretion of soluble HLA-G5 and HLA-G6 by trophoblasts (67–69). The molecular mechanism underlying this protein expression was proposed to be induced *via* the interaction of progesterone receptor complex binding to a unique progesterone response element (PRE) sequence present in the HLA-G promoter (13) (Figure 1). Classical HLA class I genes have neither a classical PRE nor the unique PRE identified in HLA-G, thus suggesting that progesterone may be involved in cell-specific regulation of the HLA-G protein expression (70, 71).

Hypoxia modulates different processes, and it is associated with induction of HLA-G. Low oxygen increases HLA-G mRNA expression in trophoblasts (72) and in tumor cell lines (73), but its effect on the expression of HLA-G protein is still undefined.

Finally, the HLA-G protein expression can be modulated by granulocyte–macrophage colony-stimulating factor (GM-CSF) in cell lines (74) and by IL-10 in monocytes (75) and DC-10 (53, 55). Although the mechanisms underlying the induction of HLA-G protein expression by the above cytokines have not been completely elucidated (Figure 1), these evidences support the role of HLA-G in promoting an anti-inflammatory and in inducting a protolerogenic microenvironment.

Overall, HLA-G protein expression is driven by specific alleles, and it is regulated by intracellular and extracellular signals. Despite results obtained in cell lines, only putative correlations between the presence of specific miRNAs and HLA-G protein levels *in vivo* have been suggested. Moreover, is has to be taken into account that the microenvironment (e.g., the presence of specific molecules, hormones and/or cytokines) might affect HLA-G protein expression. These considerations are particularly important when HLA-G genotype/protein association is studied in specific diseases.

## Concluding Remarks

The discovery that HLA-G genetic variants represent target of gene expression regulation led to intensive research for the identification of HLA-G genetic association with disease predisposition or progression and HLA-G protein expression. Despite several good genotype/protein correlations have been reported using *in vitro* model, when these observations have been translated in disease setting, the diagnostic/prognostic relevance of these findings in some cases appeared weak. A number of issue should be considered: (i) in the majority of the studies, conclusions have been based on results from small patient cohorts and in subjects from different ethnicity; (ii) only few studies performed a complete assessment of HLA-G genotyping, protein expression, and diseases outcome; (iii) the mechanisms regulating HLA-G expression can be distinctly active in different diseases; (iv) excluding the well-studied 14-bp INS/DEL polymorphism, there is a high heterogeneity of the genetic variations investigated, hindering the possibility to claim univocal conclusions.

**Figure 2:**
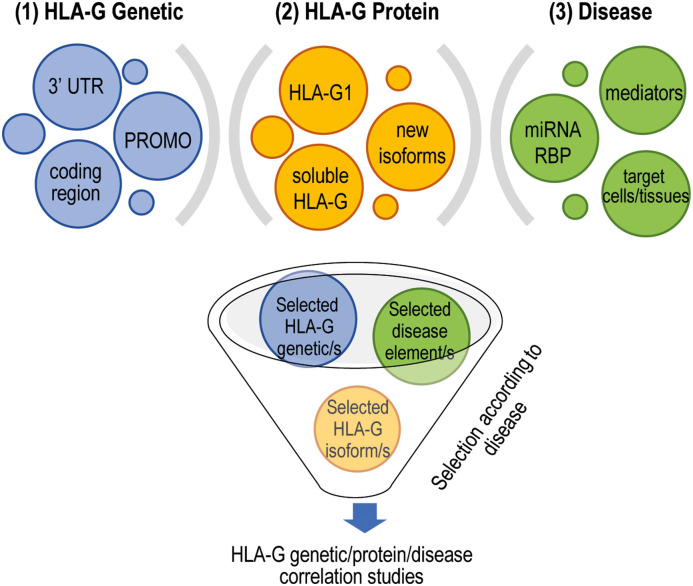
Proper selection of targets to perform HLA-G genetic/protein and disease development association studies. Different layers of complexity, both cell specific and disease specific, have to be taken into account to reliably define the role of HLA-G in disease development. (1) To select the HLA-G polymorphisms to include in the analysis; (2) to include in the analysis the evaluation of disease-specific features such as the expression of specific miRNAs or RBP and the presence of proinflammatory and anti-inflammatory mediators that can modulate the expression of HLA-G, and the target cells or tissues to study that can be affected by the presence of HLA-G; (3) to identify the most appropriate HLA-G isoforms to be investigated. 3′ UTR, 3′ untranslated region; PROMO, variations in the promoter region; miRNAs, microRNAs; RBP, RNA-binding proteins.

The specific intracellular signaling and the microenvironment characterizing a given disease have to be considered for a proper selection of the polymorphisms to be investigated. As an example, the use of the entire 3′ UTR could be relevant, but other regulatory elements (e.g., the expression of miRNAs) should be investigated in parallel, to have a comprehensive picture; otherwise, the analysis of 14-bp INS/DEL polymorphism could be sufficiently informative. Indeed, the expression of miRNAs can vary among different pathological conditions and in different cells, thus affecting the expression of HLA-G if present. Similarly, the observation that haplotypes comprising 3′ UTR, coding regions and PROMO are mainly defined by the 3′ UTR region suggested that the analysis of PROMO and coding regions of HLA-G gene should be considered for improving a better correlation between HLA-G genetic and disease predisposition if disease-specific mediators are also analyzed in parallel; otherwise, the sole analysis of the 3′ UTR might be sufficient. Finally, in several studies, the presence of HLA-G has been evaluated at mRNA but not at protein levels, being the most reliable indicator of activity. Another important aspect to consider in the analysis HLA-G proteins is the selection of the isoform, the site or cell population to be evaluated. The recent discovery in renal carcinoma cells of new HLA-G transcripts, characterized by previously not described intron retention or exon skipping events, which cannot be recognized by the available antibodies (76) increases the complexity in the field. However, whether these transcripts encode for novel isoforms, are specifically produced by cancer cells, or have regulatory functions, remain to be defined. Based on these premises, it can be envisaged that a more sophisticated selection of the parameters to be investigated is critically important to improve the HLA-G genotype/expression/disease association studies. We propose that the following steps should be taken into account: the selection of the HLA-G genetic variations (e.g., 14-bp INS/DEL or 3′ UTRs, the coding region and/or the PROMO), which might depend on the type of diseases under consideration, on the tissue or the specific subset of cells analyzed; the possibility to analyze in parallel other specific parameters (e.g., the expression of miRNAs or RBP, soluble mediators including hormones or cytokines); the selection of the most relevant HLA-G isoform (e.g., soluble HLA-Gs or HLA-G1 or other isoforms) (Figure 2).

In conclusion, up to now a clear and univocal HLA-G genotype/expression/disease association has not been yet identified, with the exception for 14-bp IND/DEL allele. A more specific and “disease-oriented” analysis, meaning the selection of the relevant polymorphisms, isoforms, and regulatory factors that could impact on HLA-G expression, would be more helpful and affordable for better defining the interplay among HLA-G genetic variations, protein expression, and disease predisposition or response to therapy.

## Data Availability Statement

The original contributions presented in the study are included in the article.

## Author Contributions

GA wrote the manuscript. SG designed and wrote the manuscript. All authors read and approve the final manuscript.

## Conflict of Interest

The authors declare that the research was conducted in the absence of any commercial or financial relationships that could be construed as a potential conflict of interest.
